# Cancer Incidence and Survival among Adolescents and Young Adults in Korea

**DOI:** 10.1371/journal.pone.0096088

**Published:** 2014-05-01

**Authors:** Eun-Kyeong Moon, Hyeon Jin Park, Chang-Mo Oh, Kyu-Won Jung, Hee Young Shin, Byung Kiu Park, Young-Joo Won

**Affiliations:** 1 Cancer Registration and Statistics Branch, National Cancer Control Institute, National Cancer Center, Goyang, Korea; 2 Cancer for Pediatric Oncology, National Cancer Center, Goyang, Korea; 3 Department of Pediatrics, Seoul National University College of Medicine, Seoul, Korea; Van Andel Institute, United States of America

## Abstract

**Background:**

In Korea, cancer is the third leading cause of death among adolescents and young adults (AYAs). However, cancer incidence and survival trends among AYAs (15–29 years) have never been studied in Korea. Therefore, this study aimed to investigate the incidence and relative survival rates and their trends among AYAs in Korea.

**Materials and Methods:**

Cancer incidence data from 1999–2010 were obtained from the Korea Central Cancer Registry (KCCR). Each cancer was classified into subgroups according to the National Cancer Institute Surveillance, Epidemiology, and End Results (SEER) AYA site recode. Percent distributions, age-specific incidence rates, age-standardized incidence rates per million, and annual percent changes (APCs) were calculated for AYAs according to sex. Five-year relative survival rates were estimated for cases diagnosed between 1993 and 2010 and followed up to 2011.

**Results:**

The age-standardized incidence rates of all cancers combined were 196.4 and 367.8 per million for males and females, respectively (male-to-female (M/F) ratio: 0.5). The age-standardized incidence rates increased from 208.7 per million in 1999 to 396.4 per million in 2010, and the APC was 6.3% (*P*<0.001). The five most common cancers among AYAs were thyroid carcinoma, non-Hodgkin lymphoma, stomach carcinoma, breast carcinoma, and acute myeloid leukemia. In males, the 5-year relative survival rate improved, from 46.5% in 1993–1995 to 75.9% in 2006–2010. In females, the 5-year relative survival rate also improved, from 66.7% in 1993–1995 to 89.1% in 2006–2010.

**Conclusions:**

Our study showed increases in cancer incidence and improvements in the 5-year relative survival rate among Korean AYAs. This study also provides additional data regarding temporal and geographic trends in cancer that may enhance future efforts to identify factors affecting cancer incidence and responses to treatment among AYAs.

## Introduction

Cancers in adolescents and young adults (AYAs; 15–29 years) have distinctive characteristics compared with cancers in children and older cohorts.

The incidence pattern of specific cancer types differ between AYAs and younger and older patients. In addition, the genetic and histologic patterns of cancers among AYAs differ from those of older patients [1]. Because cancer is uncommon among AYAs, this patient population has not drawn public attention compared with the pediatric and adult populations. However, an increase in cancer incidence among AYAs has been reported in Europe [2]–[4] and the United States [5].

Over 200,000 new cancer patients are diagnosed annually in Korea [6], and approximately 3,200 (1.6%) AYAs were diagnosed with cancer in Korea in 2010. According to the U.S. National Cancer Institute Surveillance Epidemiology and End Results (SEER), 2% of all invasive cancers are diagnosed in individuals aged 15–29 years [5]. Although only a small proportion of all malignancies are diagnosed in AYAs, high-grade and later-stage tumors of certain cancers are more likely to be diagnosed in this patient population [1]. Furthermore, cancer diagnosis in AYAs can greatly influence future quality of life and life expectancy [7]. In fact, in Korea, cancer is the leading cause of death among AYAs, after suicide and traffic accidents [8]. However, to the best of our knowledge, cancer incidence and survival among AYAs in Korea have never been studied. Therefore, this study aimed to investigate cancer incidence and survival among AYAs in Korea. We examined the trends in cancer incidence rates from 1999 to 2010 and the trends in relative survival rates from 1993 to 2010 among Korean AYAs.

## Materials and Methods

### Data Sources

In 1980, the Korean Ministry of Health and Welfare started the Korea Central Cancer Registry (KCCR), a nationwide, hospital-based cancer registry [9]. Until 1998, the registry collected cancer cases from more than 180 hospitals in Korea annually, and these data represent 80–90% of all cancer incidence in Korea [10]. Since 1999, the KCCR has covered the entire population under the population-based cancer registry program [6]. The Korea National Cancer Incidence Database (KNCIDB) KCCR data from 1999 to 2002 and from 2003 to 2007 have been published in Cancer Incidence in Five Continents, which reflects the completeness and validity of the incidence data [11].

Incidence data were collected for Korean AYAs aged 15–29 years who were newly diagnosed with cancer between 1999 and 2010. The incidence data were collected from the KNCIDB of the KCCR and included age, sex, diagnosis date, primary tumor site, morphology, the diagnostic method, and stage at diagnosis.

Survival data for individuals aged 15–29 years who were newly diagnosed with cancer from 1993–2010 were obtained from the KNCIDB, and the patients’ vital status was followed until December 31, 2011. The survival analysis was based on the KNCIDB data and mortality data obtained from Statistics Korea.

### Case Definition

In accordance with the guidelines of the National Cancer Institute SEER Program [5] and the Canadian Cancer Society [12] in this study, AYAs were defined as adolescents and young adults aged 15–29 years.

Cancer sites were coded by primary site and morphology using the International Classification of Diseases for Oncology (third edition) [13]. Cancers were classified according to the SEER AYA scheme, which is based on a modified version of the International Classification of Childhood Cancer [5]. In particular, the SEER AYA scheme is based on an updated classification proposed by Barr et al. and is composed of ten major groups and second- and third-level subgroups according to the site of origin [14].

### Incidence

Age-specific incidence rates per million were analyzed in each diagnostic subgroup according to the age at diagnosis (15–19 years, 20–24 years, and 25–29 years), and age-standardized rates (ASRs) according to sex were calculated using the world standard population defined by the World Health Organization [15]. Trends in annual ASRs were calculated using the annual percent change (APC), which was estimated using the following formula: 100×(e^β^-1), where β is the slope calculated from a linear regression of log age-standardized incidence rates in a calendar year [16]. The male-to-female (M/F) ratio was the ratio of the ASR among males to the ASR among females. Comparisons of age-standardized incidence rates in males and females were calculated by the direct method [17].

### Survival

Relative survival rates were estimated according to the time period: 1993–1995, 1996–2000, 2001–2005 and 2006–2010. Relative survival rates according to the diagnostic group were calculated by dividing observed survival by expected survival among comparable groups in the general population [18] using the Ederer II method [19]. These survival rates were estimated using “complete analysis”, which included “right-censored” patients. Due to this inclusion of the early survival experience of more recently recruited patients, the analysis provided more up-to-date and precise survival rates in long-term survival [20]. Trends in 5-year relative survival rates were also calculated. Additionally, the effects of sex, diagnostic group, and time period on survival were assessed using a relative excess risk model. All analyses were performed using SAS version 9.2.

## Results

### Incidence

From 1999 to 2010, 39,639 cancer cases (2.3% of all cancer patients) were newly diagnosed in AYAs. Of these cases, 14,016 (35.4%) and 25,623 (64.6%) cases were diagnosed in males and females, respectively. According to the age at diagnosis, 6,396 (16.1%) cases were diagnosed at 15–19 years of age, 10,433 (26.3%) cases were diagnosed at 20–24 years, and 22,810 (26.3%) cases were diagnosed at 25–29 years.

The number of cases per age group (15–19 years, 20–24 years and 25–29 years) by sex, age-specific incidence rates, and age-standardized incidence rates among both males and females and M/F ratios according to the diagnostic group are shown in Table 1.

**Table 1:** Number of cases, age-specific incidence rates, and age-standardized incidence rates from 1999 to 2010 according to sex and age.

Diagnostic group (SEER)	Males								Females								Total	M/F
	15–19 years		20–24 years		25–29 years		15–29 years		15–19 years		20–24 years		25–29 years		15–29 years			Ratio*
	Cases	CR	Cases	CR	Cases	CR	Cases	ASR	Cases	CR	Cases	CR	Cases	CR	Cases s	ASR	ASR	
**All Cancers**	**3,249**	**150.9**	**3,891**	**170.7**	**6,876**	**273.4**	**14,016**	**196.4**	**3,147**	**160.4**	**6,542**	**305.6**	**15,934**	**663.3**	**25,623**	**367.8**	**279.9**	**0.5** †
**All Cancers (excluding thyroid carcinoma)** ‡	**3,065**	**142.3**	**3,445**	**151.1**	**5,665**	**225.2**	**12,175**	**171.7**	**2,275**	**115.9**	**3,660**	**171.0**	**8,793**	**366.0**	**14,728**	**213.6**	**192.2**	**0.8** †
**1. Leukemias**	**737**	**17.1**	**600**	**13.2**	**694**	**13.8**	**2,031**	**14.8**	**482**	**12.3**	**430**	**10.0**	**549**	**11.4**	**1,461**	**11.3**	**13.1**	**1.3** †
1.1 Acute lymphoid leukemia	307	7.1	177	3.9	130	2.6	614	4.6	170	4.3	110	2.6	95	2.0	375	3.0	3.9	1.5†
1.2 Acute myeloid leukemia	265	12.3	230	10.1	287	11.4	782	11.3	226	11.5	190	8.9	283	11.8	699	10.8	11.0	1.1†
1.3 Chronic myeloid leukemia	96	4.5	131	5.7	187	7.4	414	5.8	37	1.9	75	3.5	92	3.8	204	3.0	4.5	1.9†
1.4 Other and unspecified leukemia	69	3.2	62	2.7	90	3.6	221	3.2	49	2.5	55	2.6	79	3.3	183	2.8	3.0	1.1†
**2. Lymphomas**	**516**	**24.0**	**533**	**23.4**	**612**	**24.3**	**1,661**	**23.9**	**269**	**13.7**	**414**	**19.3**	**568**	**23.6**	**1,251**	**18.7**	**21.4**	**1.3** †
2.1 Non-Hodgkin lymphoma	406	18.9	437	19.2	524	20.8	1,367	19.6	199	10.1	314	14.7	461	19.2	974	14.5	17.1	1.4†
2.2 Hodgkin lymphoma	110	5.1	96	4.2	88	3.5	294	4.3	70	3.6	100	4.7	107	4.5	277	4.2	4.3	1.0
**3. CNS and Other Intracranial and Intraspinal Neoplasms**	**332**	**15.4**	**320**	**14.0**	**406**	**16.1**	**1,058**	**15.2**	**228**	**11.6**	**214**	**10.0**	**361**	**15.0**	**803**	**12.2**	**13.8**	**1.2** †
3.1 Astrocytoma	88	4.1	110	4.8	171	6.8	369	5.2	82	4.2	74	3.5	155	6.5	311	4.7	4.9	1.1†
3.2 Other glioma	44	2.0	50	2.2	101	4.0	195	2.7	42	2.1	33	1.5	88	3.7	163	2.4	2.6	1.1†
3.3 Ependymoma	23	1.1	24	1.1	33	1.3	80	1.1	15	0.8	21	1.0	22	0.9	58	0.9	1.0	1.3†
3.4. Medulloblastoma and other PNET	66	3.1	35	1.5	24	1.0	125	1.9	47	2.4	30	1.4	24	1.0	101	1.6	1.8	1.2†
3.5 Other specified intracranial and intraspinal neoplasms	11	0.5	9	0.4	10	0.4	30	0.4	3	0.2	7	0.3	11	0.5	21	0.3	0.4	1.4†
3.6 Unspecified intracranial and intraspinal neoplasms	100	4.6	92	4.0	67	2.7	259	3.8	39	2.0	49	2.3	61	2.5	149	2.3	3.1	1.7†
**4. Osseous and Chondromatous Neoplasms**	**379**	**17.6**	**219**	**9.6**	**146**	**5.8**	**744**	**11.3**	**189**	**9.6**	**124**	**5.8**	**120**	**5.0**	**433**	**6.9**	**9.2**	**1.6** †
4.1 Osteosarcoma	257	11.9	104	4.6	53	2.1	414	6.4	126	6.4	49	2.3	47	2.0	222	3.7	5.1	1.8†
4.2 Chondrosarcoma	28	1.3	39	1.7	45	1.8	112	1.6	10	0.5	33	1.5	34	1.4	77	1.1	1.4	1.4†
4.3 Ewing tumor	53	2.5	44	1.9	18	0.7	115	1.7	30	1.5	14	0.7	21	0.9	65	1.0	1.4	1.7†
4.4 Other specified and unspecified bone tumors	41	1.9	32	1.4	30	1.2	103	1.5	23	1.2	28	1.3	18	0.7	69	1.1	1.3	1.4†
**5. Soft Tissue Sarcomas**	**220**	**10.2**	**248**	**10.9**	**345**	**13.7**	**813**	**11.5**	**174**	**8.9**	**223**	**10.4**	**302**	**12.6**	**699**	**10.5**	**11.1**	**1.1** †
5.1 Fibromatous neoplasms	44	2.0	63	2.8	92	3.7	199	2.8	35	1.8	59	2.8	88	3.7	182	2.7	2.7	1.0
5.2 Rhabdomyosarcoma	58	2.7	26	1.1	25	1.0	109	1.7	36	1.8	16	0.7	19	0.8	71	1.2	1.4	1.4†
5.3 Other soft tissue sarcoma	118	5.5	159	7.0	228	9.1	505	7.1	103	5.2	148	6.9	195	8.1	446	6.7	6.9	1.1†
**6. Germ Cell and Trophoblastic Neoplasms**	**340**	**15.8**	**417**	**18.3**	**473**	**18.8**	**1,230**	**17.6**	**341**	**17.4**	**300**	**14.0**	**295**	**12.3**	**936**	**14.7**	**16.1**	**1.2** †
6.1 Germ cell and trophoblastic neoplasms of gonads	84	3.9	231	10.1	364	14.5	679	9.3	283	14.4	243	11.4	195	8.1	721	11.4	10.3	0.8†
6.2 Germ cell and trophoblastic neoplasms of nongonadal sites	256	11.9	186	8.2	109	4.3	551	8.3	58	3.0	57	2.7	100	4.2	215	3.2	5.9	2.5†
**7. Melanoma and Skin Carcinomas**	**21**	**1.0**	**40**	**1.8**	**107**	**4.3**	**168**	**2.3**	**19**	**1.0**	**42**	**2.0**	**98**	**4.1**	**159**	**2.3**	**2.3**	**1.0**
7.1 Melanoma	12	0.6	20	0.9	50	2.0	82	1.1	11	0.6	26	1.2	46	1.9	83	1.2	1.2	0.9
7.2 Skin carcinomas	9	0.4	20	0.9	57	2.3	86	1.2	8	0.4	16	0.7	52	2.2	76	1.1	1.1	1.1
**8. Carcinomas**	**516**	**24.0**	**1,272**	**55.8**	**3,653**	**145.2**	**5,441**	**73.0**	**1,257**	**64.1**	**4,451**	**207.9**	**12,959**	**539.4**	**18,667**	**262.2**	**165.0**	**0.3** †
8.1 Thyroid carcinoma	184	8.5	446	19.6	1,211	48.1	1,841	24.7	872	44.4	2,882	134.6	7,141	297.3	10,895	154.2	87.7	0.2†
8.2 Other carcinoma of head and neck	81	3.8	133	5.8	213	8.5	427	5.9	72	3.7	133	6.2	199	8.3	404	6.0	5.9	1.0
8.2.1 Nasopharyngeal carcinoma	40	1.9	43	1.9	47	1.9	130	1.9	17	0.9	19	0.9	28	1.2	64	1.0	1.4	1.9†
8.2.2 Other sites in lip, oral cavity, and pharynx	36	1.7	81	3.6	143	5.7	260	3.6	47	2.4	104	4.9	152	6.3	303	4.4	4.0	0.8
8.2.3 Nasal cavity, middle ear, sinuses, larynx, and other ill-defined sites in head/neck	5	0.2	9	0.4	23	0.9	37	0.5	8	0.4	10	0.5	19	0.8	37	0.5	0.5	1.0
8.3 Carcinoma of trachea, bronchus, and lung	25	1.2	50	2.2	104	4.1	179	2.4	16	0.8	52	2.4	121	5.0	189	2.7	2.6	0.9†
8.4 Carcinoma of breast			2	0.1	3	0.1	5	0.1	15	0.8	265	12.4	1,672	69.6	1,952	26.5	13.0	0.003†
8.5 Carcinoma of genitourinary tract	30	1.4	93	4.1	290	11.5	413	5.5	154	7.8	524	24.5	1,986	82.7	2,664	37.1	20.9	0.1†
8.5.1 Carcinoma of kidney	17	0.8	52	2.3	173	6.9	242	3.2	18	0.9	34	1.6	108	4.5	160	2.3	2.8	1.4†
8.5.2 Carcinoma of bladder	12	0.6	30	1.3	103	4.1	145	1.9	2	0.1	16	0.7	31	1.3	49	0.7	1.3	2.8†
8.5.3 Carcinoma of gonads	-	-	3	0.1	2	0.1	5	0.1	123	6.3	242	11.3	420	17.5	785	11.5	5.6	0.01†
8.5.4 Carcinoma of cervix and uterus	-	-	-	-	-	-	-	-	8	0.4	229	10.7	1,399	58.2	1,636	22.2	10.8	-
8.5.5 Carcinoma of other and ill-defined sites in genitourinary tract	1	0.0	8	0.4	12	0.5	21	0.3	3	0.2	3	0.1	28	1.2	34	0.5	0.4	0.6†
8.6 Carcinoma of gastrointestinal tract	161	7.5	500	21.9	1,715	68.2	2,376	31.5	113	5.8	553	25.8	1,740	72.4	2,406	33.5	32.5	0.9†
8.6.1 Carcinoma of colon and rectum	67	3.1	176	7.7	530	21.1	773	10.3	46	2.3	165	7.7	402	16.7	613	8.7	9.5	1.2†
8.6.2 Carcinoma of stomach	41	1.9	179	7.9	764	30.4	984	12.9	33	1.7	298	13.9	1,116	46.5	1,447	19.9	16.3	0.6†
8.6.3 Carcinoma of liver and intrahepatic bile ducts	51	2.4	121	5.3	373	14.8	545	7.3	18	0.9	66	3.1	151	6.3	235	3.3	5.4	2.2†
8.6.4 Carcinoma of pancreas	1	0.0	16	0.7	21	0.8	38	0.5	12	0.6	14	0.7	32	1.3	58	0.9	0.7	0.6†
8.6.5 Carcinoma of other and ill-defined sites in gastrointestinal tract	1	0.0	8	0.4	27	1.1	36	0.5	4	0.2	10	0.5	39	1.6	53	0.7	0.6	0.6†
8.7 Carcinoma of other and ill-defined sites	35	1.6	48	2.1	117	4.7	200	2.7	15	0.8	42	2.0	100	4.2	157	2.2	2.5	1.2†
8.7.1 Adrenocortical carcinoma	1	0.0	5	0.2	6	0.2	12	0.2	3	0.2	7	0.3	8	0.3	18	0.3	0.2	0.6†
8.7.2 Carcinoma of other and ill-defined sites, NOS	34	1.6	43	1.9	111	4.4	188	2.6	12	0.6	35	1.6	92	3.8	139	2.0	2.3	1.3†
**9. Miscellaneous Specified Neoplasms, NOS**	**120**	**5.6**	**117**	**5.1**	**181**	**7.2**	**418**	**6.0**	**100**	**5.1**	**141**	**6.6**	**254**	**10.6**	**495**	**7.3**	**6.6**	**0.8** †
9.1 Other pediatric and embryonal tumors, NOS	30	1.4	20	0.9	24	1.0	74	1.1	20	1.0	17	0.8	22	0.9	59	0.9	1.0	1.2†
9.2 Other specified and embryonal tumors, NOS	90	4.2	97	4.3	157	6.2	344	4.9	80	4.1	124	5.8	232	9.7	436	6.4	5.6	0.8†
**10. Unspecified Malignant Neoplasms**	**67**	**3.1**	**125**	**5.5**	**259**	**10.3**	**451**	**6.2**	**88**	**4.5**	**203**	**9.5**	**428**	**17.8**	**719**	**10.4**	**8.2**	**0.6** †

*M/F Ratio  =  Male ASR/Female ASR.

† P-values <0.05.

‡ Thyroid carcinoma was excluded from the calculation of the incidence rate of all cancers combined because of its unusually high incidence rate.

CR, crude incidence rate; CNS, central nervous system; PNET, primitive neuroectodermal tumor; NOS, not otherwise specified.

Between 1999 and 2010, the overall age-standardized incidence rate of cancers among AYAs in Korea was 279.9 per million. Cancer incidence was higher in females (367.8 per million) than in males (196.4 per million), and for all cancers combined, the male/female ratio was 0.5 (*P*<0.05). The higher rate among females was largely due to a much higher incidence rate of thyroid carcinomas (24.7 per million among males vs. 154.2 per million among females). Because the incidence rate of thyroid carcinoma was unusually high, the age-standardized incidence rates of all cancers combined were recalculated, excluding thyroid carcinoma (group 8.1). Removing thyroid carcinomas, the overall ASR of cancers was 192.2 per million (171.7 per million for males and 213.6 per million for females) (Table 1).

The incidence increased with age, from 150.9 in males and 160.4 in females per million at 15–19 years of age to 170.7 in males and 305.6 in females per million, respectively, at 20–24 years of age. The incidence further increased at 25–59 years of age, to 273.4 in males and 663.3 in females per million, respectively (Table 1). The incidence rates were correlated with age group for most subtypes, with the notable exceptions of leukemia and osseous/chondromatous neoplasms, which were more common among younger AYAs (Table 1).

**Figure 1:**
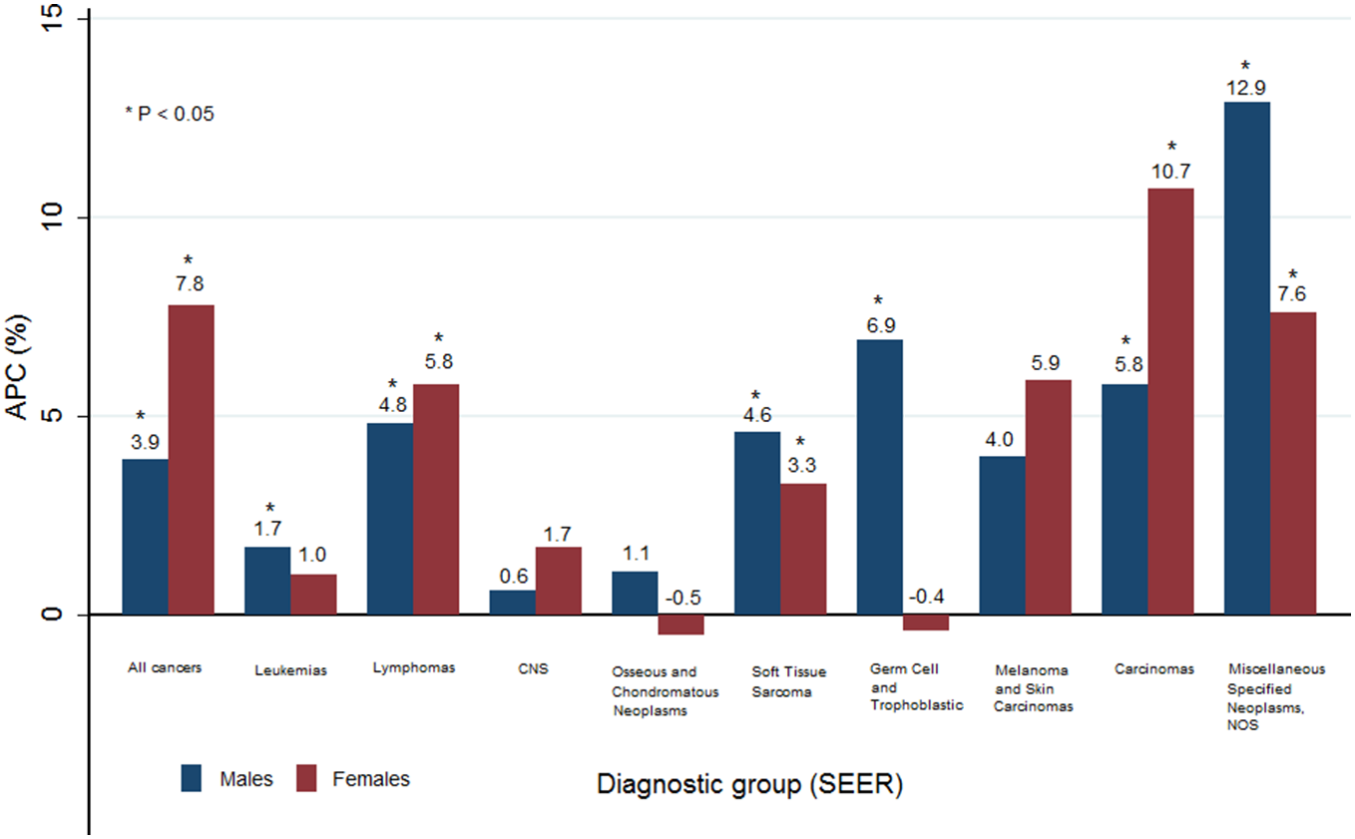
Annual percent change (APC) from 1999–2010 according to the diagnostic group (SEER).

Table 2 shows the secular trends in cancer incidence among AYAs from 1999 to 2010 according to the diagnostic group. The incidence rate of all cancers among AYAs significantly increased, from 208.7 per million in 1999 to 396.4 per million in 2010 (APC = 6.3%; *P*<0.05). Over the studied time period, there was also a steady increase in the incidence of cancer among AYAs for both males (APC = 3.9%) and females (APC = 7.8%) (Figure 1).

**Table 2:** Trends in age-standardized incidence rates among Korean AYAs and estimated annual percent changes (APCs).

	1999	2000	2001	2002	2003	2004	2005	2006	2007	2008	2009	2010	APC
All cancers	208.7	204.7	230.3	226.4	256.3	266.2	275.6	290.8	315.8	355.6	370.4	396.4	6.3*
All cancers (excluding thyroid carcinoma) †	174.8	170.9	188.2	178.9	193.6	188.0	193.5	197.1	203.8	210.7	208.2	207.5	1.8*
**1 Leukemias**	23.4	24.1	27.8	26.0	24.5	24.5	25.6	26.6	28.2	29.6	28.9	26.5	1.5*
1.1 Acute lymphoid leukemia	7.0	7.6	7.4	7.2	6.7	6.9	7.7	8.0	8.4	8.3	8.9	8.8	2.2*
1.2 Acute myeloid leukemia	10.4	9.2	12.3	12.2	10.5	10.5	10.8	12.5	11.3	12.7	11.1	9.3	0.3
1.3 Chronic myeloid leukemia	3.5	4.3	4.7	3.7	4.7	4.4	4.5	3.6	4.9	5.0	5.3	5.5	2.9*
1.4 Other and unspecified leukemia	2.5	3.0	3.4	2.8	2.7	2.6	2.7	2.5	3.6	3.5	3.6	2.8	1.5
**2 Lymphomas**	16.6	15.0	17.7	17.2	21.8	23.1	21.5	23.9	23.9	26.7	26.3	26.0	5.3*
2.1 Non-Hodgkin lymphoma	14.5	12.5	14.4	13.6	17.2	18.2	17.7	19.6	18.6	20.7	20.8	19.9	4.5*
2.2 Hodgkin lymphoma	2.1	2.4	3.3	3.6	4.6	4.9	3.8	4.3	5.3	6.0	5.5	6.1	9.1*
**3 CNS and Other Intracranial and Intraspinal Neoplasms (all behaviors)**	12.7	12.9	12.0	13.4	15.0	14.5	12.9	14.6	14.5	15.7	14.1	13.3	1.2
3.1. Astrocytoma	4.8	3.8	4.7	4.5	4.7	5.4	4.6	5.0	6.3	5.6	4.8	5.3	2.3*
3.2 Other glioma	1.4	1.8	1.5	2.8	2.8	2.5	2.5	2.6	2.7	4.2	3.3	3.4	8.0*
3.3 Ependymoma	0.5	1.0	1.1	0.9	1.1	0.9	0.7	1.2	1.0	1.4	1.2	1.3	4.9*
3.4. Medulloblastoma and other PNET	1.6	1.5	1.6	2.0	2.3	2.3	1.2	2.4	1.3	2.0	2.0	1.1	-0.8
3.5 Other specified intracranial and intraspinal neoplasms	0.6	0.4	0.4	0.2	0.5	0.4	0.3	0.2	0.5	0.5	0.2	0.3	-3.9
3.6 Unspecified intracranial and intraspinal neoplasms	3.7	4.5	2.8	3.0	3.7	2.9	3.6	3.1	2.8	2.0	2.5	2.1	-5.0*
**4 Osseous & Chondromatous Neoplasms**	8.4	8.7	11.4	7.9	10.6	8.5	8.2	7.6	9.7	10.2	8.8	10.5	0.6
4.1 Osteosarcoma	5.0	4.9	6.8	4.9	5.6	5.3	4.9	3.8	4.4	6.1	4.4	5.2	-1.1
4.2 Chondrosarcoma	1.4	1.4	1.1	0.9	1.9	1.0	1.3	1.1	1.5	1.6	1.2	2.0	2.3
4.3 Ewing tumor	0.7	1.3	1.7	1.2	1.4	1.0	1.2	1.3	1.9	1.3	1.9	2.1	5.8*
4.4 Other specified and unspecified bone tumors	1.3	1.1	1.8	0.9	1.7	1.1	0.8	1.5	1.9	1.2	1.3	1.2	0.4
**5 Soft Tissue Sarcomas**	8.9	8.5	10.6	9.5	11.0	10.9	9.7	12.5	13.0	12.9	13.6	12.8	4.1*
5.1 Fibromatous neoplasms	1.4	1.8	1.7	2.0	3.2	2.7	2.5	3.3	3.2	4.5	3.1	4.2	9.6*
5.2 Rhabdomyosarcoma	1.2	1.6	1.2	1.6	0.6	2.5	1.3	2.4	1.5	0.7	1.0	1.3	-1.6
5.3 Other soft tissue sarcoma	6.3	5.0	7.7	5.9	7.2	5.7	5.9	6.8	8.3	7.7	9.4	7.3	3.2*
**6 Germ Cell and Trophoblastic Neoplasms**	13.5	14.0	14.8	14.3	14.5	14.7	17.0	16.4	19.1	17.8	18.3	20.2	3.6*
6.1 Germ cell and trophoblastic neoplasms of gonads	9.1	8.6	9.9	9.0	9.0	10.2	10.3	10.3	11.2	12.0	11.4	12.9	3.3*
6.2 Germ cell and trophoblastic neoplasms of nongonadalsites	4.4	5.5	4.9	5.3	5.6	4.4	6.7	6.1	8.0	5.9	7.0	7.3	4.2*
**7 Melanoma and Skin Carcinomas**	1.7	2.1	2.6	1.8	1.0	1.5	3.7	2.5	2.3	2.7	3.2	2.5	4.8
7.1 Melanoma	1.1	1.2	1.2	0.9	0.5	0.9	2.0	0.9	0.9	1.4	1.9	1.1	2.6
7.2 Skin carcinomas	0.6	0.8	1.5	0.9	0.6	0.5	1.7	1.6	1.4	1.3	1.4	1.5	7.2*
**8 Carcinomas**	105.3	102.0	116.4	123.2	141.5	155.2	163.7	173.1	191.8	225.1	244.4	270.7	9.4*
8.1 Thyroid carcinoma	33.9	33.8	42.1	47.4	62.6	78.2	82.1	93.6	112.0	144.9	162.2	188.9	17.9*
8.2 Other carcinoma of head and neck	4.9	5.1	5.0	5.9	6.7	5.9	6.1	5.3	6.8	6.7	8.1	5.2	2.5
8.2.1 Nasopharyngeal carcinoma	1.1	1.8	1.2	1.2	1.9	1.6	1.3	1.1	1.4	1.7	1.8	1.4	1.2
8.2.2 Other sites in lip, oral cavity and pharynx	3.2	3.0	3.3	4.2	3.9	3.8	4.4	3.9	5.0	4.4	5.5	3.6	3.4*
8.2.3 Nasal cav,mid ear,sinuses,larynx,oth ill-defhead/neck	0.6	0.4	0.4	0.5	0.9	0.6	0.4	0.3	0.5	0.7	0.7	0.3	-0.6
8.3 Carcinoma of trachea,bronchus, and lung	2.4	1.5	2.9	2.2	2.5	3.0	3.1	3.0	1.9	2.2	2.9	3.4	2.6
8.4 Carcinoma of breast	8.5	11.4	12.3	13.6	12.2	12.6	15.1	13.8	13.9	14.1	13.8	15.3	3.5*
8.5 Carcinoma of genitourinary tract	18.4	14.7	17.2	18.7	21.5	21.1	20.9	23.8	22.2	24.5	24.0	25.7	4.2*
8.5.1 Carcinoma of kidney	2.2	0.9	2.3	2.1	2.1	2.8	2.5	4.2	2.4	4.3	4.4	3.3	9.1*
8.5.2 Carcinoma of bladder	1.4	0.8	1.4	1.1	1.7	1.5	1.8	1.1	1.6	1.2	0.9	1.4	0.7
8.5.3 Carcinoma of gonads	5.9	5.9	4.9	5.5	6.8	5.2	5.8	5.2	6.4	4.9	4.4	6.1	-0.8
8.5.4 Carcinoma of cervix and uterus	8.6	6.7	8.4	9.5	10.3	11.1	10.3	13.0	11.3	13.9	13.7	14.6	6.2*
8.5.5 Carc of other and ill-def sites, geniourinary tract	0.2	0.3	0.2	0.5	0.5	0.4	0.5	0.3	0.5	0.2	0.5	0.3	2.8
8.6 Carcinoma of gastrointestinal tract	34.2	32.3	34.2	33.1	34.3	31.6	34.0	31.2	31.7	31.1	31.1	30.4	-1.0*
8.6.1 Carcinoma of colon and rectum	9.1	8.1	8.4	7.7	9.1	8.3	9.2	9.4	9.8	12.9	11.8	11.3	3.7*
8.6.2 Carcinoma of stomach	18.8	17.3	18.4	18.7	17.4	15.8	17.3	15.4	15.3	13.2	13.7	13.2	-3.3*
8.6.3 Carcinoma of liver and intrahepatic bile ducts	5.2	5.5	6.8	5.8	6.3	5.4	6.0	5.2	5.3	3.6	4.3	4.5	-3.2*
8.6.4 Carcinoma of pancreas	0.4	0.8	0.3	0.6	0.6	1.0	0.8	0.8	0.8	0.6	0.4	1.1	4.8
8.6.5 Carc other and ill-def sites, gastrointestinal tract	0.7	0.6	0.4	0.2	0.8	1.0	0.6	0.4	0.6	0.8	1.0	0.3	0.1
8.7 Carcinoma of other and ill-def sites	3.0	3.3	2.7	2.3	1.8	2.8	2.4	2.5	3.1	1.7	2.4	2.0	-3.0
8.7.1 Adrenocortical carcinoma	0.2	0.2	0.1	0.2	0.2	0.1	0.1	0.4	0.5	0.1	0.4	-	6.7
8.7.2 Carcinoma of other and ill-defined sites, NOS	2.8	3.0	2.6	2.1	1.6	2.7	2.3	2.1	2.6	1.6	1.9	2.0	-3.4*
**9 Miscellaneous specified neoplasms, NOS**	3.1	4.4	4.3	4.3	7.5	6.4	7.5	8.2	8.2	9.7	8.1	9.1	9.8*
9.1 Other pediatric and embryonal tumors, NOS	0.7	1.3	0.7	0.8	1.4	0.6	1.3	0.9	0.9	1.5	0.7	1.4	3.0
9.2 Other specified and embryonal tumors, NOS	2.5	3.1	3.6	3.5	6.0	5.8	6.3	7.4	7.3	8.3	7.4	7.7	11.3
**10 Unspecified Malignant Neoplasms**	15.1	13.1	12.7	8.8	8.8	7.1	5.6	5.3	5.0	5.1	4.6	4.7	-10.9*

*P-values <0.05.

† Thyroid carcinoma was excluded from the calculation of the incidence rate of all cancers combined because of its unusually high incidence rate.

AYAs, adolescents and young adults (aged 15–29 years); CNS, central nervous system; PNET, primitive neuroectodermal tumor; NOS, not otherwise specified.

Most cancer sites showed a trend of increasing incidence, with the exception of unspecified malignant neoplasms (group 10) among AYAs (APC  =  −10.9%). For miscellaneous specified neoplasms, NOS (group 9), a rapid increase in the incidence rate was observed for both sexes (APC = 9.8%), but the number of cases was small. For carcinomas (group 10), a large increase in the incidence rate was also observed among AYAs (APC = 9.4%). In particular, among carcinomas, the incidence of thyroid carcinoma showed the most rapid increase (APC = 17.9%; *P*<0.05). The annual percent change in all cancer combined, excluding thyroid carcinoma, was 1.8% (*P<*0.05) among AYAs (Table 2), with 1.9% (*P*<0.05) for males and 1.8% (*P*<0.05) for females (data not shown). Although the incidence of kidney carcinoma also exhibited a notable increase (APC = 9.1%), the number of cases was small.

The incidence of lymphomas (group 2) exhibited a large increase among AYAs (APC = 5.3%, *P*<0.05). In particular, Hodgkin lymphoma rapidly increased in incidence (APC = 9.1%, *P*<0.05) (Table 2).

Cancer incidence trends within diagnostic groups were observed to differ by gender. Among males, the incidence of most cancer sites was increased. Miscellaneous specified neoplasms, NOS, showed the largest increase in incidence (APC = 12.9%, *P*<0.05), followed by germ cell and trophoblastic neoplasms (APC = 6.9%, *P*<0.05). Among females, the incidence of most cancer sites was also increased. Carcinomas (APC  = 10.7%, *P*<0.05) and miscellaneous specified neoplasms, NOS (APC = 7.6%, *P*<0.05), showed the greatest increases. However, osseous/chondromatous neoplasms (APC  =  −0.5%) and germ cell and trophoblastic neoplasms (APC  =  −0.4%) slightly decreased in incidence (Figure 1). Additionally, a notable increase in the incidence of carcinomas among females was observed in the cervix (APC = 6.2%, *P*<0.05) and breast (APC = 3.5%, *P*<0.05) (data not shown).

### Survival

A total of 52,077 cancer cases diagnosed from 1993 to 2010 were used for the survival analysis. Table 3 shows the 5-year relative survival rates and numbers of cases in the four time periods (1993–1995, 1996–2000, 2001–2005 and 2006–2010). For all cancers combined, the 5-year relative survival rate of AYAs significantly improved, from 58.9% in 1993–1995 to 84.8% in 2006–2010 (*P*<0.05). AYAs with leukemia and lymphoma showed the most marked improvement in survival from 25.8% (95% CI: 22.9–28.7) and 55.4% (95% CI: 50.6–59.9) in 1993–1995 to 58.8% (95% CI: 55.5–61.8) and 83.6% (95% CI: 80.9–85.9) in 2006–2010, respectively. Conversely, decreases in survival were observed from 1993–1995 to 2006–2010 for other glioma (group 3.2), carcinoma of gonads (group 8.5.3), and carcinoma of pancreas (group 8.6.4) (Table 3).

**Table 3:** Five-year RSRs for Korean AYAs according to the time period of cancer diagnosis**.**

Diagnostic group (SEER)	Both sexes								Change†	*P*
	1993–1995		1996–2000		2001–2005		2006–2010			
	Cases	RSR	Cases	RSR	Cases	RSR	Cases	RSR		
**All Cancers**	**6,387**	**58.9**	**12,453**	**66.4**	**14,310**	**76.5**	**18,927**	**84.8**	**25.9**	*
**All Cancers (excluding thyroid carcinoma)** ‡	**5,525**	**52.6**	**10,474**	**60.2**	**10,555**	**68.2**	**10,884**	**74.4**	**21.8**	*
**1. Leukemias**	**866**	**25.8**	**1,440**	**35.9**	**1,397**	**47.7**	**1,435**	**58.8**	**33.0**	*
1.1 Acute lymphoid leukemia	237	19.9	433	29	379	33.9	425	48.6	28.7	*
1.2 Acute myeloid leukemia	375	26.0	602	37.5	624	47.2	588	52.7	26.7	*
1.3 Chronic myeloid leukemia	141	41.4	251	51.2	252	76	262	90.5	49.1	*
1.4 Other and unspecified leukemia	113	17.8	154	24.1	142	36	160	58.8	41.0	*
**2. Lymphomas**	**439**	**55.4**	**937**	**65**	**1,133**	**75.8**	**1,315**	**83.6**	**28.2**	*
2.1 Non-Hodgkin lymphoma	372	51.3	790	60.6	906	72.5	1,037	82.0	30.7	*
2.2 Hodgkin lymphoma	67	78.0	147	88.8	227	88.8	278	89.4	11.4	*
**3. CNS and Other Intracranial and Intraspinal Neoplasms**	**371**	**53.4**	**689**	**54.5**	**730**	**60**	**750**	**65.6**	**12.2**	*
3.1. Astrocytoma	179	43.2	305	45.1	275	45.2	291	54.3	11.1	*
3.2 Other glioma	65	72.7	83	61.7	141	66.1	177	71.2	-1.5	
3.3 Ependymoma	14	86.1	49	79.9	57	88.0	62	87.0	0.9	
3.4 Medulloblastoma and other PNET	18	44.6	84	49	92	47.9	84	52.3	7.7	
3.5 Other specified intracranial and intraspinal neoplasms	13	61.9	27	74.4	20	80.2	18	72.6	10.7	
3.6 Unspecified intracranial and intraspinal neoplasms	82	55.2	141	61.2	145	76.1	118	80.6	25.4	*
**4. Osseous and Chondromatous Neoplasms**	**262**	**48.3**	**516**	**66.1**	**479**	**68.7**	**447**	**74.8**	**26.5**	*
4.1 Osteosarcoma	160	43.3	305	65.5	281	65.3	231	72.2	28.9	*
4.2 Chondrosarcoma	28	64.6	75	87.0	69	87.2	77	94.9	30.3	*
4.3 Ewing tumor	30	40.2	66	39.5	67	52.4	72	51.0	10.8	
4.4 Other specified and unspecified bone tumors	44	61.7	70	71.7	62	80.9	67	85.5	23.8	*
**5. Soft Tissue Sarcomas**	**243**	**58.7**	**481**	**58.8**	**554**	**67.5**	**617**	**73.4**	**14.7**	*
5.1 Fibromatous neoplasms	51	78.8	103	78	135	93.6	193	95.1	16.3	*
5.2 Rhabdomyosarcoma	32	28.3	73	37.1	66	34.9	55	37.5	9.2	
5.3 Other soft tissue sarcoma	160	58.4	305	57.6	353	63.6	369	67.8	9.4	*
**6. Germ Cell and Trophoblastic Neoplasms**	**278**	**81.6**	**745**	**87**	**842**	**89.9**	**936**	**91.7**	**10.1**	*
6.1 Germ cell and trophoblastic neoplasms of gonads	174	89.4	465	92.3	558	94.3	613	96.2	6.8	*
6.2 Germ cell and trophoblastic neoplasms of nongonadal sites	104	68.6	280	78.1	284	81.2	323	83.2	14.6	
**7. Melanoma and Skin Carcinomas**	**56**	**64.6**	**111**	**59.7**	**122**	**77.3**	**138**	**86.3**	**21.7**	*
7.1 Melanoma	25	32.2	64	43.9	63	60.5	59	65.0	32.8	*
7.2 Skin carcinomas	31	90.8	47	81.2	59	95.2	79	99.0	8.2	*
**8. Carcinomas**	**3,205**	**67.5**	**6,607**	**72.2**	**8,366**	**82.4**	**12,601**	**89.9**	**22.4**	*
8.1 Thyroid carcinoma	862	99.3	1,979	99.6	3,755	99.9	8,043	99.9	0.6	*
8.2 Other carcinoma of head and neck	140	69.6	289	77.8	339	83.1	333	85.5	15.9	*
8.2.1 Nasopharyngeal carcinoma	40	70.4	104	65.7	79	80.0	75	85.1	14.7	*
8.2.2 Other sites in lip, oral cavity, and pharynx	73	79.8	159	87.7	233	85.6	235	88.6	8.8	
8.2.3 Nasal cavity, middle ear, sinuses,larynx, and other ill-defined sites in head/neck	27	41.0	26	65.6	27	70.6	23	60.5	19.5	
8.3 Carcinoma of trachea, bronchus, and lung	73	24.8	136	36.2	144	47.4	132	43.1	18.3	*
8.4 Carcinoma of breast	310	68.0	691	77.2	825	82.0	841	86.5	18.5	*
8.5 Carcinoma of genitourinary tract	602	85.3	1,191	85.5	1,194	88.9	1,371	86.5	1.2	
8.5.1 Carcinoma of kidney	35	68.9	94	76.9	142	87.6	209	87.0	18.1	*
8.5.2 Carcinoma of bladder	39	85.1	77	94.0	90	93.6	73	95.9	10.8	
8.5.3 Carcinoma of gonads	234	86.6	396	84.5	326	88.2	291	82.7	-3.9	
8.5.4 Carcinoma of cervix and uterus	285	86.6	605	86.7	611	89.1	781	87.0	0.4	
8.5.5 Carcinoma of other and ill-defined sites,genitourinary tract	9	78.3	19	79.2	25	84.2	17	88.1	9.8	
8.6 Carcinoma of gastrointestinal tract	1,123	38.0	2,127	42.3	1,974	50.2	1,766	59.2	21.2	*
8.6.1 Carcinoma of colon and rectum	246	47.0	527	52.8	500	63.2	603	73.9	26.9	*
8.6.2 Carcinoma of stomach	723	36.3	1,239	41.4	1,091	49.0	831	58.2	21.9	*
8.6.3 Carcinoma of liver andintrahepatic bile ducts	90	23.5	277	23.6	308	33.6	256	31.6	8.1	*
8.6.4 Carcinoma of pancreas	30	56.9	35	57.3	39	59.1	42	43.8	-13.1	
8.6.5 Carcinoma of other and ill-defined sites ingastrointestinal tract	34	29.6	49	45.1	36	39.0	34	59.1	29.5	*
8.7 Carcinoma of other and ill-defined sites	95	41.3	194	39.3	135	44.6	115	54.0	12.7	*
8.7.1 Adrenocortical carcinoma	N/S									
8.7.2 Carcinoma of other and ill-defined sites, NOS	88	40.0	181	39.4	128	46.2	99	56.2	16.2	*
**9. Miscellaneous Specified Neoplasms, NOS**	**121**	**54.0**	**213**	**72.5**	**348**	**79.2**	**453**	**79.7**	**25.7**	*
9.1 Other pediatric and embryonaltumors, NOS	33	30.5	43	46.7	51	57.0	51	63.1	32.6	*
9.2 Other specified and embryonal tumors, NOS	88	62.8	170	79.1	297	83.0	402	81.7	18.9	*
**10. Unspecified Malignant Neoplasms**	**546**	**62.0**	**714**	**70.8**	**339**	**79.0**	**235**	**77.8**	**15.8**	*

*P-values <0.05 for trend.

† Change (%) in the 5-year RSR from 1993–1995 to 2006–2010.

‡ Thyroid carcinoma was excluded from the calculation of the incidence rate of all cancers combined because of its unusually high incidence rate.

N/S: not shown because <20 cases were reported in each period.

AYAs, adolescents and young adults (aged 15–29 years); CNS, central nervous system; PNET, primitive neuroectodermal tumor; NOS, not otherwise specified.

Survival rates for thyroid carcinoma (group 8.1) and skin carcinoma (group 7.2) were very high across all time periods. The five-year relative survival rate for thyroid carcinoma among males increased slightly, from 95.3% in 1993–1995 to 99.7% in 2006–2010, whereas the rate was unchanged among females from 1993–1995 (99.9%) to 2006–2010 (100.0%). The survival rates for germ cell and trophoblastic neoplasms (group 6), skin carcinoma (group 7.2), and carcinoma of the genitourinary tract (group 8.5) consistently exceeded 80–90% in all time periods. Conversely, the lowest survival rates were observed for rhabdomyosarcoma (group 5.2); carcinoma of the trachea, bronchus, and lung (group 8.3); and carcinoma of liver and intrahepatic bile ducts (group 8.6.3) (Table 3).

The survival rates for all cancers combined significantly increased from 1993 to 2010 in both males and females. In particular, the 5-year relative survival rate increased from 46.5% to 75.9% in males (*P*<0.05) and from 66.7% to 89.1% in females (*P*<0.05). However, the 5-year relative survival rate for all cancers combined was slightly lower in males than in females, regardless of whether thyroid carcinoma was excluded (Table 4).

**Table 4:** Five-year RSRs for Korean AYAs according to the time period of cancer diagnosis and sex.

Diagnostic group (SEER)	Males										Females									
	1993–1995		1996–2000		2001–2005		2006–2010		Change†	*P*	1993–1995		1996–2000		2001–2005		2006–2010		Change†	*P*
	Cases	RSR	Cases	RSR	Cases	RSR	Cases	RSR			Cases	RSR	Cases	RSR	Cases	RSR	Cases	RSR		
**All Cancers**	**2,478**	**46.5**	**4,801**	**55.1**	**5,166**	**65.8**	**6,149**	**75.9**	**29.4**	*	**3,909**	**66.7**	**7,652**	**73.6**	**9,144**	**82.6**	**12,778**	**89.1**	**22.4**	*
**All Cancers (excluding thyroid carcinoma)** ‡	**2,366**	**44.2**	**4,516**	**52.4**	**4,696**	**62.3**	**4,924**	**70.5**	**26.3**	*	**3,159**	**58.9**	**5,958**	**66.0**	**5,859**	**72.9**	**5,960**	**77.6**	**18.7**	*
**1. Leukemias**	**492**	**25.4**	**791**	**33.0**	**826**	**46.2**	**841**	**57.9**	**32.5**	*	**374**	**26.3**	**649**	**39.4**	**571**	**49.8**	**594**	**60.0**	**33.7**	*
1.1 Acute lymphoid leukemia	140	22.3	253	27.4	236	30.2	272	49.2	26.9	*	97	16.5	180	31.2	143	39.9	153	47.4	30.9	*
1.2 Acute myeloid leukemia	194	26.5	307	30.4	318	44.5	313	51.1	24.6	*	181	25.5	295	44.8	306	50.1	275	54.7	29.2	*
1.3 Chronic myeloid leukemia	87	40.5	152	51.6	183	74.0	173	89.1	48.6	*	54	42.7	99	50.6	69	81.3	89	93.2	50.5	*
1.4 Other and unspecified leukemia	71	9.9	79	25.5	89	37.2	83	51.4	41.5	*	42	31.0	75	22.7	53	34.0	77	67.3	36.3	*
**2. Lymphomas**	**262**	**50.7**	**558**	**60.9**	**637**	**72.8**	**748**	**80.9**	**30.2**	*	**177**	**62.3**	**379**	**71.1**	**496**	**79.6**	**567**	**87.0**	**24.7**	*
2.1 Non-Hodgkin lymphoma	230	47.3	480	56.7	522	70.2	601	79.2	31.9	*	142	57.9	310	66.6	384	75.7	436	85.9	28.0	*
2.2 Hodgkin lymphoma	32	75.5	78	86.3	115	84.6	147	87.3	11.8	*	35	80.2	69	91.5	112	93.0	131	91.3	11.1	*
**3. CNS and Other Intracranial and Intraspinal Neoplasms**	**220**	**49.9**	**393**	**52.2**	**417**	**59.4**	**423**	**63.0**	**13.1**	*	**151**	**58.4**	**296**	**57.6**	**313**	**60.8**	**327**	**68.9**	**10.5**	*
3.1. Astrocytoma	104	36.8	169	39.8	156	43.1	155	48.4	11.6		75	52.2	136	51.6	119	48.0	136	60.8	8.6	
3.2 Other glioma	41	76.1	53	56.9	68	70.8	102	66.9	-9.2		24	66.9	30	70.2	73	61.8	75	77.0	10.1	
3.3 Ependymoma	5	100.6	32	84.8	28	86.0	39	94.0	-6.6		9	78.0	17	70.7	29	89.8	23	74.6	-3.4	
3.4 Medulloblastoma and other PNET	8	37.8	43	44.4	51	37.4	46	41.5	3.7		10	50.1	41	53.8	41	61.1	38	63.8	13.7	
3.5 Other specified intracranial and intraspinal neoplasms	N/S										N/S								-	
3.6 Unspecified intracranial and intraspinal neoplasms	54	54.1	80	61.5	102	77.7	72	78.3	24.2	*	28	57.3	61	60.8	43	72.2	46	84.3	27.0	*
**4. Osseous and Chondromatous Neoplasms**	**163**	**48.8**	**317**	**65.0**	**290**	**66.1**	**296**	**74.7**	**25.9**	*	**99**	**47.6**	**199**	**68.0**	**189**	**72.6**	**151**	**75.4**	**27.8**	*
4.1 Osteosarcoma	106	42.7	195	64.4	174	60.5	160	71.4	28.7	*	54	44.6	110	67.4	107	73.0	71	74.6	30.0	*
4.2 Chondrosarcoma	17	71.1	43	86.5	44	93.5	44	93.5	22.4		11	54.7	32	87.7	25	76.2	33	96.4	41.7	
4.3 Ewing tumor	15	47.0	43	37.4	38	44.9	46	49.7	2.7		15	33.4	23	43.6	29	62.2	26	53.3	19.9	
4.4 Other specified and unspecified bone tumors	25	60.4	36	75.4	34	82.6	46	89.4	29.0	*	19	63.3	34	67.8	28	78.7	21	77.1	13.8	
**5. Soft Tissue Sarcomas**	**126**	**52.7**	**244**	**57.7**	**287**	**63.3**	**342**	**68.8**	**16.1**	*	**117**	**65.1**	**237**	**60.1**	**267**	**72.1**	**275**	**78.6**	**13.5**	*
5.1 Fibromatous neoplasms	23	78.8	61	75.8	64	90.9	103	93.4	14.6	*	28	78.8	42	81.1	71	96.0	90	96.7	17.9	*
5.2 Rhabdomyosarcoma	20	25.2	43	35.0	41	34.2	35	34.1	8.9		12	33.4	30	40.1	25	36.1	20	44.9	11.5	
5.3 Other soft tissue sarcoma	83	52.2	140	56.7	182	60.1	204	62.8	10.6	*	77	65.1	165	58.3	171	67.4	165	73.4	8.3	
**6. Germ Cell and Trophoblastic Neoplasms**	**98**	**72.9**	**323**	**79.3**	**457**	**85.8**	**574**	**88.8**	**15.9**	*	**180**	**86.4**	**422**	**92.9**	**385**	**94.7**	**362**	**96.3**	**9.9**	*
6.1 Germ cell and trophoblastic neoplasms of gonads	47	90.0	157	85.8	255	90.5	331	95.4	5.4	*	127	89.2	308	95.7	303	97.5	282	97.0	7.8	*
6.2 Germ cell and trophoblastic neoplasms of nongonadal sites	51	57.2	166	73.2	202	79.9	243	79.9	22.7		53	79.5	114	85.3	82	84.3	80	93.6	14.1	
**7. Melanoma and Skin Carcinomas**	**24**	**71.3**	**66**	**57.9**	**66**	**73.0**	**67**	**83.9**	**12.6**	*	**32**	**59.6**	**45**	**62.4**	**56**	**82.3**	**71**	**89.1**	**29.5**	*
7.1 Melanoma	11	45.8	38	45.0	33	51.7	26	45.0	-0.8		14	21.5	26	42.4	30	70.1	33	76.5	55.0	*
7.2 Skin carcinomas	13	93.0	28	75.4	33	94.3	41	97.9	4.9		18	89.2	19	89.7	26	96.4	38	100.2	11.0	
**8. Carcinomas**	**872**	**52.3**	**1,787**	**57.0**	**1,935**	**68.2**	**2,561**	**80.5**	**28.2**	*	**2,333**	**73.1**	**4,820**	**77.9**	**6,431**	**86.6**	**10,040**	**92.4**	**19.3**	*
8.1 Thyroid carcinoma	112	95.3	285	97.3	470	100.2	1,225	99.7	4.4	*	750	99.9	1,694	99.9	3,285	99.8	6,818	100.0	0.1	
8.2 Other carcinoma of head and neck	76	62.3	147	71.1	177	79.4	170	86.8	24.5	*	64	78.4	142	84.7	162	87.2	163	84.5	6.1	
8.2.1 Nasopharyngeal carcinoma	28	71.9	70	60.3	56	80.6	48	91.6	19.7	*	12	66.9	34	76.7	23	78.4	27	77.6	10.7	
8.2.2 Other sites in lip, oral cavity, and pharynx	32	72.4	65	86.6	108	79.9	112	86.6	14.2		41	85.6	94	88.5	125	90.5	123	90.3	4.7	
8.2.3 Nasal cavity, middle ear, sinuses, larynx, and other ill-defined sites in head/neck	N/S										N/S									
8.3 Carcinoma of trachea, bronchus, and lung	41	24.6	68	37.0	65	52.5	64	39.6	15.0		32	25.1	68	35.4	79	43.1	68	46.7	21.6	*
8.4 Carcinoma of breast	N/S										309	67.9	686	77.3	822	81.9	838	86.6	18.7	*
8.5 Carcinoma of genitourinary tract	52	83.3	116	91.0	158	91.5	197	92.2	8.9		550	85.5	1,075	85.0	1,036	88.5	1,174	85.5	0	
8.5.1 Carcinoma of kidney	14	79.2	43	86.5	85	89.8	129	91.9	12.7		21	62.1	51	68.8	57	84.4	80	78.8	16.7	*
8.5.2 Carcinoma of bladder	30	87.3	61	95.6	61	95.4	60	95.0	7.7		N/S									
8.5.3 Carcinoma of gonads	N/S										231	86.8	392	84.4	324	88.1	290	82.6	-4.2	
8.5.4 Carcinoma of cervix and uterus	N/S										285	86.6	605	86.7	611	89.1	781	87.0	0.4	
8.5.5 Carcinoma of other and ill-defined sites in genitourinary tract	N/S										N/S									
8.6 Carcinoma of gastrointestinal tract	536	43.1	1,064	43.0	984	49.4	842	56.5	13.4	*	587	33.3	1,063	41.5	990	51.0	924	61.7	28.4	*
8.6.1 Carcinoma of colon and rectum	140	51.1	299	50.1	287	62.6	330	73.6	22.5	*	106	41.6	228	56.3	213	64.0	273	74.6	33.0	*
8.6.2 Carcinoma of stomach	307	44.0	530	48.0	445	51.9	310	56.5	12.5	*	416	30.6	709	36.5	646	47.0	521	59.3	28.7	*
8.6.3 Carcinoma of liver and intrahepatic bile ducts	62	26.0	193	19.3	226	28.4	170	31.4	5.4	*	28	17.9	84	33.4	82	47.7	86	31.4	13.5	
8.6.4 Carcinoma of pancreas	N/S										21	66.9	18	83.5	24	71.0	28	69.3	2.4	
8.6.5 Carcinoma other and ill-defined sites in gastrointestinal tract	18	28.0	25	44.3	11	45.6	18	45.3	17.3		16	31.3	24	46.0	25	36.1	16	75.2	43.9	*
8.7 Carcinoma of other and ill-defined sites	54	31.7	102	44.3	78	51.5	60	48.1	16.4	*	41	53.8	92	33.8	57	35.2	55	60.2	6.4	
8.7.1 Adrenocortical carcinoma	N/S										N/S									
8.7.2 Carcinoma of other and ill-defined sites, NOS	51	33.6	94	44.9	76	51.5	53	48.7	15.1	*	37	48.8	87	33.4	52	38.5	46	66.1	17.3	
**9. Miscellaneous Specified Neoplasms, NOS**	**51**	**49.4**	**80**	**52.8**	**154**	**71.0**	**221**	**81.0**	**31.6**	*	**70**	**57.3**	**133**	**84.4**	**194**	**85.7**	**232**	**78.6**	**21.3**	
9.1 Other pediatric and embryonal tumors, NOS	21	33.6	24	33.5	32	53.3	27	62.6	29.0	*	12	25.1	19	63.3	19	63.3	24	63.4	38.3	
9.2 Other specified and embryonal tumors, NOS	30	60.4	56	61.0	122	75.6	194	83.4	23.0	*	58	64.0	114	87.9	175	88.2	208	80.3	16.3	
**10. Unspecified Malignant Neoplasms**	**170**	**40.9**	**242**	**56.1**	**97**	**64.2**	**76**	**70.7**	**29.4**	*	**376**	**71.5**	**472**	**78.4**	**242**	**84.9**	**159**	**81.3**	**9.8**	*

*P-values <0.05 for trend.

† Change (%) in the 5-year RSRs from 1993–1995 to 2006–2010.

‡ Thyroid carcinoma was excluded from the incidence rate of all cancers combined because of its unusually high incidence rate.

N/S: not shown because <20 cases were reported in each period.

AYAs, adolescents and young adults (aged 15–29 years); CNS, central nervous system; PNET, primitive neuroectodermal tumor; NOS, not otherwise specified.

Leukemia (group 1) showed the greatest increase in survival in both males (32.5%) and females (33.7%). In particular, chronic myeloid leukemia had the largest and second-largest increases in survival in males (48.6%; from 40.5% to 89.1%) and females (50.5%; from 42.7% to 93.2%), respectively.

**Figure 2:**
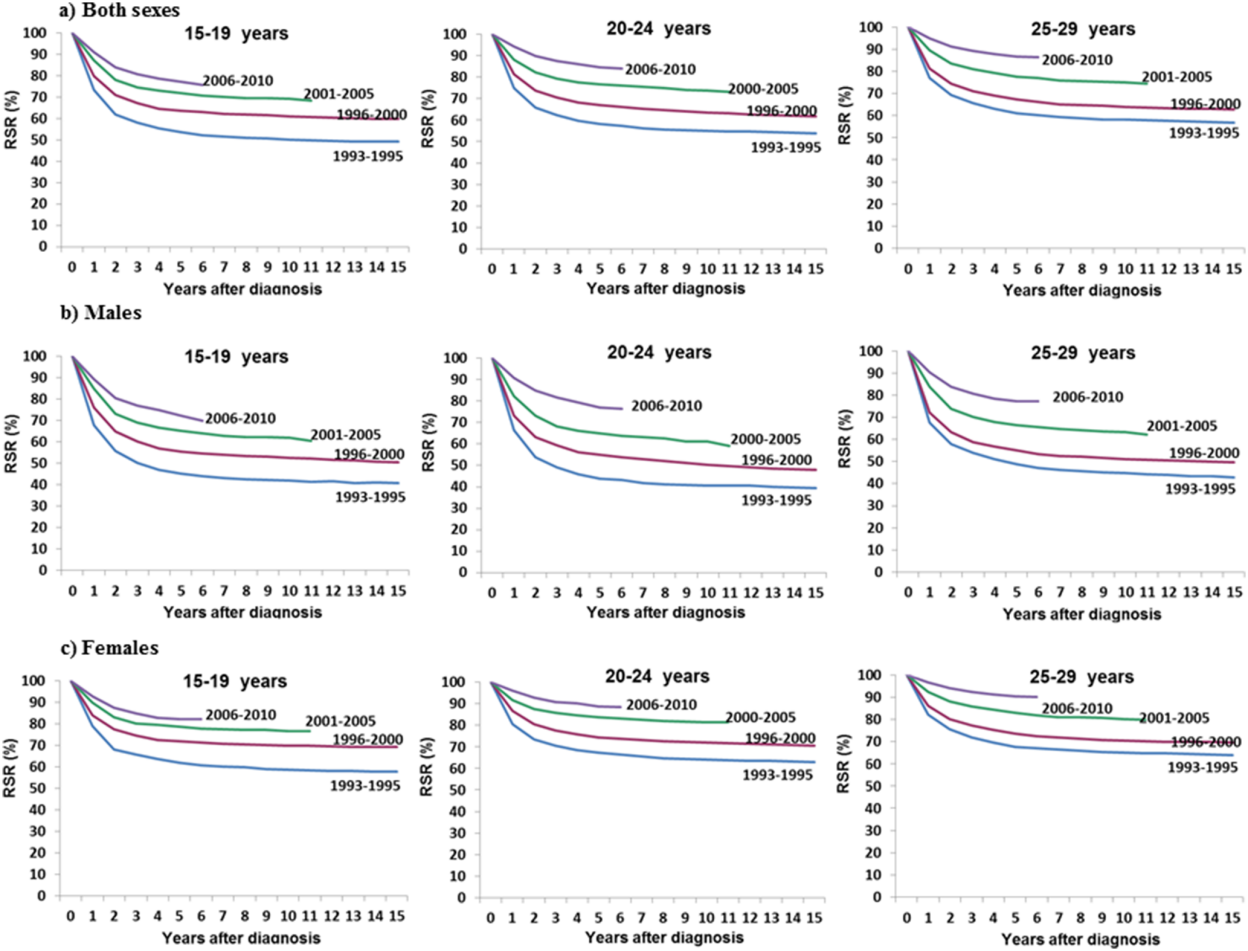
Trends in relative survival after cancer in Korea according to age and the time period.

Figure 2 depicts the 5-year relative survival rates of all cancer patients in each of the four time periods according to age (15–19 years, 20–24 years and 25–29 years) and sex. Both gender, the 5-year relative survival rates increased in all age groups. For males aged 15–19 years, the 5-year relative survival rates in 1993–1995, 1996–2000, 2001–2005, and 2006–2010 for all cancers combined were 45.3% (95.% CI: 41.2–49.2), 55.4% (95% CI: 52.5–58.1), 65.3% (95% CI: 62.5–67.9), and 72.2% (95% CI: 69.2–75.0), respectively. The survival rates of males aged 20–24 years were 43.9% (95% CI: 40.2–47.5), 55.0% (95% CI: 52.2–57.7), 65.0% (95% CI: 62.5–67.3), and 77.0% (95% CI: 74.3–79.5) in 1993–1995, 1996–2000, 2001–2005, and 2006–2010, respectively. The survival rates of males aged 25–29 years were 48.8% (95% CI: 45.9–51.7), 55.0% (95% CI: 52.9–57.0), 66.5% (95% CI: 64.6–68.3), and 77.2% (95% CI: 75.3–79.0) in 1993–1995, 1996–2000, 2001–2005, and 2006–2010, respectively. For females aged 15–19 years, the 5-year relative survival rates for all cancers combined were 62.0% (95% CI: 58.0–65.8), 72.0% (95% CI: 69.4–74.5), 78.8% (95% CI: 76.3–81.0), and 82.2% (95% CI: 79.7–84.5) in 1993–1995, 1996–2000, 2001–2005, and 2006–2010, respectively. The survival rates of females aged 20–24 years were 67.4% (95% CI: 64.6–70.1), 74.4% (95% CI: 72.4–76.3), 83.7% (95% CI: 82.2–85.1), and 88.9% (95% CI: 87.4–90.2) in 1993–1995, 1996–2000, 2001–2005, and 2006–2010, respectively. The survival rates of females aged 25–29 years were 67.7% (95% CI: 65.7–69.6), 73.6% (95% CI: 72.2–74.9), 82.9% (95% CI: 81.9–83.9), and 90.4% (95% CI: 89.5–91.2) in 1993–1995, 1996–2000, 2001–2005, and 2006–2010, respectively.

## Discussion

This is the first study to investigate cancer incidence, survival and their trends among AYAs using the population-based national cancer registry in Korea. The major findings of this study were that cancer in AYAs showed a trend of increasing incidence, with an increase of 6.3% per year (*P*<0.05), from 1999 to 2010 and that age- and gender-related cancer incidence patterns differed according to the primary site. Moreover, five-year relative survival rates for most cancers improved from 1993–1995 (58.9%) to 2006–2010 (84.8%) among AYAs.

When comparing our study with studies from other countries, cancer incidence rates among AYAs in our study were lower than incidence rates in the U.S. [5], France [21], Portugal [22], and Netherlands [3] and among males in Canada [12], even though the time period and age group differs. In other studies, cancer incidence in AYA males was generally similar to or higher than cancer incidence in AYA females. Conversely, we reported much lower incidence rates in males than in females. The reason for this difference in the incidence rate by gender was that thyroid carcinoma has the highest incidence and rapidly increased in incidence among AYA females in Korea.

Consistent with other studies, we found a rising incidence of cancer among AYAs during the study period.

The data on AYAs in Korea reported here exhibited several important differences from site-specific cancer incidence rates among AYAs in other regions of the world. Since the 2000s, an annual increase in incidence of 0.6–2.0% has been reported in several countries [16], [23]–[25]. However, our results showed an annual increase in incidence of 6.3%, which is a more rapid increase than observed in other studies. The increased cancer incidence rate may be partially explained by changes in cancer classification, as exemplified by changes in the classification of hematologic malignancies in a study by Park et al. [26].

The increased incidence of carcinomas was primarily due to an increase in the incidence rates of thyroid carcinoma (APC = 17.9%). An increased incidence rate of thyroid carcinoma has also been noted among AYAs in Western countries [23], [27], [28]. However, the incidence of thyroid carcinoma among AYAs is more than three- to tenfold higher in Korea than in Canada [12], England [23], the United States [5], the Netherlands [3] and Portugal [22]. The reasons for the high incidence of thyroid carcinoma in Korean AYAs compared with other nationalities are unknown. Although the rapid increase and high incidence rate of thyroid cancer among older individuals worldwide might be attributable to the development of improved technologies for early detection [29], the exact cause of the increased incidence of most cancers in AYAs is unknown. Because of the difficulty in recommending thyroid cancer screening for AYAs solely based on incidence rates, further research to identify associated risk factors, such as family history, socioeconomic status, and environmental exposure, is needed.

In this study, a notable trend of increasing incidence was also observed for cervical carcinoma (APC = 6.2%) among female AYAs in Korea. Although the incidence of cervical carcinoma in Korean females of all ages is decreasing (APC  =  −4.3%) [6], the incidence of cervical carcinoma has been increasing among Korean females under 30 years of age [30]. A steady increase in cervical carcinoma in young women (20–29 years) has also been observed in England [31]. The increased incidence of cervical carcinoma among AYAs has been attributed to increases in human papillomavirus (HPV) infection [32], [33]. More specifically, an increase in sexual behavior among younger age groups has led to an increased rate of HPV infection [34], [35], and the prevalence rate of HPV has been reported to increase with decreasing age [32]. Therefore, since 2007, the Korean Society of Gynecologic Oncology and Colposcopy (KSGOC) has recommended the HPV vaccine for females aged 15–17 for the prevention of cervical carcinoma. In fact, certain recent studies have reported a decrease in the incidence of cervical carcinoma due to the use of the HPV vaccine at an earlier age [36], [37]. Therefore, the incidence of cervical carcinoma is expected to gradually decline among AYAs in Korea due to the HPV vaccine.

In terms of survival, our data are consistent with that reported for other geographic regions. Although the time period in our study differed, the overall cancer survival rate among AYAs in Korea was similar to the rate and significantly improvement reported in the U.S. and Germany. Improvements in relative survival rates among AYAs can be partially explained by advances in cancer detection, more intensive treatments, and increased expertise in adolescent oncology [38]. Additionally, access to effective protocols and the development of health infrastructures may have also contributed to improvements in survival rates [39].

However several important differences should be highlighted in lymphoma and leukemia. In the present study, the most significant improvements in survival were observed in leukemia and lymphoma patients, but the survival rates for leukemia and lymphoma were noticeably lower than in the U.S. and Germany [40], [41]. This reason for this difference in the survival rate by ethnic was that the incidence cases of subgroup of leukemia and lymphoma was different between U.S. AYAs [41] and Korea AYAs. More, ethnic disparities in tumor biology and clinical factors may influence cancer treatment and survival [42].

Compared with the survival of patients aged 1 to 10 years, overall survival and disease-specific survival are clinically significantly poorer among AYA patients with acute lymphoblastic leukemia [43]. The survival rates for leukemia among the Korean AYAs in our study have remained worse than among Korean children based on data from the KCCR [44].

Among AYAs, breast cancer accounts for approximately 7% and 4.9% of all cancers diagnosed in the United States [5] and Korea, respectively. In the United States, the 5-year survival rate for breast cancer is lower among AYAs (80.2%) than among patients in other age groups (30–39 years, 83.4%; 40–49 years, 88.9%), and particularly older patients [41]. Our study showed similar results. The relative survival rate for breast cancer among Korean females aged 15–29 years was 86.8% in 2006–2010, whereas the relative survival rate among Korean females aged ≥40 years was 91.0% based on a direct estimate from the KNCIDB.

One limitation of our study is that the follow-up period began relatively soon after the diagnosis of cancer, in contrast to the protocols in other studies [3]–[5]. Another limitation of this study is that we could not estimate the survival rates after adjusting for cancer stage and treatment because our registry database did not include information on cancer stage and treatment.

In conclusion, our study provides representative cancer statistics regarding temporal trends in the AYA population in Korea. In particular, the results showed an increasing trend in cancer incidence and an improving survival trend among AYAs in Korea. These results may support cancer control and prevention plans focusing on AYAs.

In the future, further research will help to identify factors affecting cancer incidence and responses to treatment among AYAs. In particular, research on the etiological factors related to the rapid increase in thyroid carcinoma in AYAs is needed.
